# I’m wearing a mask, but are they?: Perceptions of self-other differences in COVID-19 health behaviors

**DOI:** 10.1371/journal.pone.0269625

**Published:** 2022-06-06

**Authors:** James Adaryukov, Sergej Grunevski, Derek D. Reed, Timothy J. Pleskac

**Affiliations:** 1 Brain, Behavior, and Quantitative Sciences Program, Psychology Department, University of Kansas, Lawrence, Kansas, United States of America; 2 Applied Behavioral Sciences Department, University of Kansas, Lawrence, Kansas, United States of America; 3 Cofrin Logan Center for Addiction Research and Treatment, Lawrence, Kansas, United States of America; Texas A&M University, UNITED STATES

## Abstract

As information about COVID-19 safety behavior changed, people had to judge how likely others were to protect themselves through mask-wearing and vaccination seeking. In a large, campus-wide survey, we assessed whether University of Kansas students viewed others’ protective behaviors as different from their own, how much students assumed others shared their beliefs and behaviors, and which individual differences were associated with those estimations. Participants in our survey (*N* = 1, 704; 81.04% white, 64.08% female) estimated how likely they and others were to have worn masks on the University of Kansas campus, have worn masks off-campus, and to seek a vaccine. They also completed measures of political preference, numeracy, and preferences for risk in various contexts. We found that participants estimated that others were less likely to engage in health safety behaviors than themselves, but that their estimations of others were widely shared. While, in general, participants saw themselves as more unique in terms of practicing COVID-19 preventative behaviors, more liberal participants saw themselves as more unique, while those that were more conservative saw their own behavior as more similar to others. At least for masking, this uniqueness was false—estimates of others’ health behavior were lower than their actual rates. Understanding this relationship could allow for more accurate norm-setting and normalization of mask-wearing and vaccination.

## Introduction

People commonly misestimate the characteristics of those around them. For instance, people tend to misestimate demographic statistics (i.e. “How many people in your township do you think are Muslim?”) and rates of engaging in harmful behavior (i.e. “How many people in your township do you think habitually smoke?”) in their own neighborhoods [1]. The potential for these misestimations seems especially relevant during the COVID-19 pandemic. Rapid updating of COVID-related information, particularly during the early stages of the pandemic, led to frequent changes in safety guidelines. These changes, in turn, resulted in uneven adoption of protective behavior in populations [2], communities [3], and even individuals [4]. Because of this unevenness, people were often left on their own to judge how likely others were to protect themselves from COVID-19, the number of people that had the illness, or how effective treatments were [5]. This is just as true in the present moment, as vaccination efforts have also involved rapidly updating information on their effectiveness and public adoption [6].

Two oft-cited psychological explanations for these misestimations are the *false consensus* and the *false uniqueness effects*. In a false consensus effect, individuals perceive the behaviors of a most available representative—typically the self—as common in their own group or the general population, and behaviors in which they do not engage as less common [7, 8]. For instance, if they judge themselves as likely to use their high-beam headlights while driving, they treat that behavior as representative and estimate that others also use their high-beams. First examined in the context of conflict resolution [7], this phenomenon has since been found to apply to practiced behaviors [9–12] and held beliefs [13, 14], including in health-specific contexts. For instance, in the case of health behavior, [15] found that those who assumed others in their social in-group were vaccinated were more likely to vaccinate themselves. The converse effect, false uniqueness, occurs when individuals underestimate the prevalence of their most available representative’s behaviors or beliefs in the general population (e.g., estimate others as less likely to use their high-beams) [16, 17]. Sometimes false consensus and false uniqueness can be observed in the same context. For instance, respondents in a study by [18] both overestimated how many people shared their aversion to crowded social events (false consensus) and underestimated people’s emotional reactions to said crowded events (false uniqueness). In other words, they thought most others would try to avoid the crowds but would be less urgent in that avoidance.

Whether people display false consensus or false uniqueness depends on several factors in their environment. When making social estimates, people often base their estimates on their own in-group or a group which contains that in-group [19], such that more homogeneous groups tend to display more false consensus [20]. For instance, wealthy individuals whose social circles are also wealthy think that the general population is wealthier [21]. If within-group likeness (i.e., homophily) is reduced by, for instance, groups becoming more tolerant of other groups or seeing others in the group as dissimilar to them, then estimates of behavior and belief tend toward false uniqueness instead [22–24]. In addition, false consensus manifests when individuals feel isolated or under threat, such as when they face social rejection or believe their behaviors are being judged negatively [8, 25]. Those whose beliefs or behaviors are more socially normative or desirable, meanwhile, tend to see them as more unique than they actually are [26]. Finally, salience plays a role in individual consensus and uniqueness, with people more likely to contrast from themselves if their own attitudes are brought to mind first [27]. Taken together, the implication is that both perceived difference and social evaluation of that difference as positive or negative influence consensus vs. uniqueness in estimation.

False consensus and false uniqueness are especially relevant to individual judgments of COVID-19 because of their impacts on behavioral *intent*. Perceived consensus can encourage the continuation of even obviously harmful behaviors, such as smoking [9, 28]. Such attitudes can spill over into policy support; participants in [21] who saw the public as wealthier supported welfare policy less. Both effects have dire implications for health intervention. An analysis of National Flu Survey data conducted before COVID-19 [15] found that people who believed those in their group would not vaccinate showed less intent to seek vaccines themselves (see also [29]). The troubling implication is that motivated false consensus could reduce willingness to adopt protective behavior and inspire others to do the same, leading to fewer and fewer people protecting themselves from illness. Furthermore, people who perceive their behavior as falsely unique may not speak out about their beliefs, or even shift their own attitudes to what they perceive as the majority [30, 31]. In the current context, this means that if those who are protecting themselves see themselves in the minority, they may not convey that to others, or might even reduce their protective behavior. Both forms of misestimation could interfere with individual response to COVID-19. Therefore, our study sought to answer two questions:

***Q1***: *Do differences exist between one’s willingness to engage in COVID-19-related health behavior and their perceptions of others’ willingness to engage in the same behavior (i.e., self-other differences)?*
***Q2***: *Are these perceived self-other differences consistent with the false consensus or false uniqueness effect?*

To address these questions, we had student participants report on their rates of on-campus and off-campus mask wearing, as well as their intent to get vaccinated. These were all evaluated on percent likelihood scales, from 0% likely to 100% likely. We then asked participants to estimate how likely other students were to engage in the same behaviors on equivalent scales. For on-campus mask-wearing, we also compared student estimations to direct observational data of students’ masking behaviors.

Although we asked students to estimate for a general “other”, if COVID-19 has brought one realization to the forefront, it is that people’s responses to and beliefs about the pandemic seemed to vary quite wildly based on demographic factors. For instance, Black, Latino, and Asian Americans were more likely to wear masks [32] and less likely to seek vaccines [33]; a similar pattern was present for women of all racial backgrounds [33]. People’s risk preferences were also associated with their likelihood to get vaccinated [34]. Finally, political orientation was associated with COVID-19 behaviors and beliefs. People with a more conservative political preference expressed less intention to seek a vaccine [33], a lower likelihood of wearing masks while outside [4], and more frequent cases of “never” wearing masks [35].

Some of these differences are consistent with factors that impact the false consensus effect. For instance, false consensus manifests more when comparing oneself to the same gender [36] and the same overall political orientation [17, 37]. Thus, we also use this study to investigate a third question:

***Q3***: *What individual differences are associated with perceived self-other differences and to what degree?*

To investigate this question, we collected measures of race, gender, riskiness, and political orientation, alongside other salient individual difference measures such as numeracy and discounting (preference for immediate vs. future rewards). We correlated these measures with participants’ estimates of themselves, their estimates of others, and the differences between their self and other estimates.

## Materials and methods

All data were collected from a university-wide survey conducted at the University of Kansas from December 3 to December 17, 2020. For context, at the time, the university required masks on campus in any spaces used by multiple people, indoors and outdoors. Masks were specifically defined as “a covering of the nose and mouth” [38]. The University of Kansas held classes (in person and virtual) up until the Thanksgiving holiday (November 26 and 27, 2020). Following the holiday, students were instructed to not return to campus after the holiday and instructors provided a final project for students to complete. Vaccines were authorized for emergency use beginning December 11, although only Pfizer vaccines were available until December 18th, after the study had concluded. Rollout plans at the University of Kansas had not been formalized. Public conversation surrounding the vaccines at the time was largely positive, with many seeing them as rays of hope against the pandemic [39]. However, some concern persisted that the vaccines were rushed through development [40].

### Participants and demographics

An invitation went out to all 19,135 undergraduate students enrolled at the University of Kansas in the fall of 2020. A total of 2,659 students started the university-wide survey. Of these students, 1,704 (64.08%) completed all relevant measures and were included in the final analysis. The average sample age was 21 years old (SD = 5.37). Across the sample, 61.4 percent of respondents identified as female, 34.6 percent identified as male, and 4.0 percent identified as nonbinary or did not specify their gender. A total of 1,381 respondents (81.04%) identified as White, 208 (12.21%) identified as Asian, and 69 (4.05%) identified as Black or African-American. In terms of ethnicity, a total of 168 participants (9.86%) identified as Hispanic or Latino. In addition, a total of 86 (5%) of the respondents were international students.

The research protocol was approved by the University of Kansas Institutional Review Board. Before taking the survey, consent was obtained for each participant online. Consent forms were not linked to survey responses and no personally identifying information was collected during the study.

### Materials

#### COVID-19 behavior items

*Likelihood judgments*. Three COVID-19-related health behaviors were assessed in the survey: wearing masks while on-campus, wearing masks while off-campus, and seeking a vaccine. For mask-wearing, participants were asked to estimate what percentage of time they wore a mask on any given day within the two weeks before the Thanksgiving holiday (November 10, 2020, to November 24, 2020), specifically between 12:00 and 3:00 PM (peak times of student activity between classes), on-campus and off-campus. As mentioned earlier, class meetings ended after the Thanksgiving holiday, with students only working on final projects. Thus, this period reflected the last two weeks people were in and around campus. Only participants who had indicated that they had been present on-campus and in the vicinity of Lawrence were included in the calculations. In addition to estimating their own rates of mask-wearing, participants were asked to estimate what percentage of time other students wore masks on campus and off-campus during the same time block. The survey did not specify whether masks had to cover one’s nose and mouth.

For vaccination, as the survey was conducted before vaccines were widely available, participants were asked to estimate how likely they would be to seek a COVID-19 vaccine when approved by the FDA and widely available. They were also asked to estimate how likely others would be to seek a COVID-19 vaccine at that time. Because of skew in our overall distributions of answers across outcomes, outcome measures were transformed on a log-odds scale before analysis. Differences were measured by subtracting self-estimates for each outcome measure by estimates of others after log-odds transformation.

*Consensus*. As part of this study, we also asked participants the following question: “Imagine that 100 people were asked the following question: ‘On any given day during the time period, what percentage of the time do you think others wore a mask on campus?’ How do you believe they would respond?”. To answer this question, participants were asked to estimate how many students (out of 100) they believed would answer at eleven different level brackets of percentages: 0% to 4%, 5% to 9%, … 95% to 100%. We also asked the same question about wearing masks off-campus and the likelihood of seeking a vaccine. We initially collected this data for a different project to predict COVID-19 health behaviors among students using the Surprisingly Popular algorithm [41]. These results will be reported elsewhere. Here, we use these responses to estimate the degree of false consensus for attitudes via a method adapted from [7]. Participants’ judgments were assigned to levels of our discretized scale based on their responses (i.e. those who estimated others as 65% likely to wear masks were matched with the 60–69% bracket). As a measure of consensus, we used the relative proportion of students that the participants thought would respond with a similar judgment. For example, if a respondent estimated that others wore a mask on campus 90% of the time, the measure of consensus was what proportion of students the respondents thought would answer between 85% and 94%.

#### Direct observational data

In addition to student estimates, trained undergraduate research assistants collected on-campus mask-wearing data from November 12, 2021 to November 23, 2020 (the same time point our survey respondents were asked to consider) at 6 locations across campus. Observers recorded mask-wearing for a total of 1,136 individuals. Observers recorded the number of individuals with a mask fully covering the nose and mouth, as well as the number of individuals without full coverage; we converted these data into proportions of students compliant with the mask-wearing policy. Across all observations, 89.94% of individuals were observed in full compliance with the mask policy, with 93.70% masked in some capacity (i.e., wearing a mask regardless of whether it fully covered the nose and mouth). Because the survey did not specify the degree of compliance, we focused our analyses on the proportion of individuals masked in any capacity.

#### Individual difference measures

*Political preference*. Political preference is particularly notable as a predictor in this study due to its demonstrated associations both with false consensus [37] and willingness to engage in health and safety measures [42, 43]. Furthermore, older [27] and more recent [17, 44] studies have connected political identity (liberal vs. conservative) to both false consensus and false uniqueness, with liberals displaying more false uniqueness and conservatives displaying more false consensus. Participants in this study reported their own political preference on a single-item scale of 1 to 11, from “Extremely Liberal” to “Extremely Conservative”.

*Numeracy*. Risk perception is often associated with numeracy, or an intuitive familiarity with and understanding of numbers; the more numerate an individual is, the more precise their evaluations of risks in given behavior [45]. Numeracy is also associated with an individual’s overall predictive propensity and quality of judgment [46]. Therefore, we asked participants to complete a short four-question version of the Berlin Numeracy Scale, or BNS [47]. The Berlin Numeracy Scale evaluates both the accuracy and granularity of individuals’ probability judgments in multiple contexts. Participants responded by selecting from one of four answer choices (including “None of these”), and could use scratch paper and notes, but no calculator. An example question is, “In a forest, 20 percent of mushrooms are red, 50 percent brown and 30 percent white. A red mushroom has a 20 percent probability of being poisonous. A mushroom that is not red has a 5 percent probability of being poisonous. What is the probability that a poisonous mushroom in the forest is red?”. Participants were evaluated on a scale of 1 to 5, with each correct answer being marked as 1 and each incorrect answer being marked as 0; scores were summed across questions. One point was added to participant scales to avoid any total scores of 0. In our sample, the Cronbach’s alpha for this scale was 0.399 [0.353, 0.444]; while low, this is consistent with earlier uses of the scale, which reported similarly low alphas [45].

*Riskiness*. According to the seminal Health Behavior Model, decisions involving personal health involve cost-benefit analyses—is the cost of seeking intervention or adopting protective behavior worth avoiding the consequences of illness? [48]—and risk judgments—how risky is the illness which I am trying to avoid? [49, 50]. Therefore, risk perception and associated measures play a significant role in deciding health behavior, both in terms of risk of the illness and risk of the intervention [49, 51] with more risk-loving individuals less likely to engage in health-protective behavior [52]. Therefore, we asked participants to complete self-report scales of riskiness. For riskiness, participants were asked to report how risky they are in various domains on a scale of 1, “Not risky at all”, to 11, “Extremely risky.” Riskiness was assessed for recreational, driving, health-related, financial, occupational, social, and overall domains. In our sample, the Cronbach’s alpha for the riskinesss scale was 0.841 [0.829, 0.853], indicating strong internal reliability. For the purposes of this study, we focused on the single-item overall domain of riskiness.

*Discounting task*. Health behavior overall is associated with discounting behavior, or the ability to wait longer for greater rewards as opposed to settling for lesser rewards in the short term [53]. Focusing on short-term benefits, indicated by greater discounting of future options, correlates with increased risky health behaviors [54] and decreased adherence to existing treatment [55]. Thus, participants completed a 5-trial adjusting delay assessment of discounting [56]. In each trial, participants were asked to choose between an immediate $50 reward or a $100 reward after a larger amount of time (both are hypothetical rewards). For instance, “would you prefer $50 now or $100 in two weeks?” This rapid adjusting delay procedure titrates the delay to the $100 across 5 trials to arrive at an indifference point (i.e., the delay associated with indifference between an immediate $50 and a delayed $100). This indifference point is considered the “effective delay” for 50% discounting (*ED*_50_), the inverse of which yields the discounting parameter *k* [57]. The logarithm of the *k* score was taken and used as the outcome measure, with higher log values indicating greater preference for immediate options.

### Analysis

We first examined zero-order correlations between our outcome measures and predictive factors. Next, Bayesian multilevel regressions were performed for all outcomes. To evaluate self-other differences, regressions were performed with the magnitude of estimation as an outcome variable and target of estimation (self or other) and behavior measured as categorical predictors. To measure associations between individual differences and health behavior, additional Bayesian regressions were run in R’s Rstanarm package with default priors [58]. A total of 4 chains were compiled per regression, with 10,000 iterations per chain (5,000 of which were burn-in). 20,000 sample values were drawn from the posterior distribution of each iteration. Rhat values for each variable had converged to approximately 1.00 by the time the regressions had been run. We worked to reach an effective sample size of at least 10,000 on each parameter.

Three models were compared per outcome measure. In the *politics and behavioral economics model*, political preference, riskiness, numeracy, and discounting (expressed by the log of the K score) were included as predictors. In the *politics and race set*, only political preferences and race were included as predictors. Finally, the *full model* included all predictors. The credible intervals for each predictor were found to be similar across all models; therefore, results from the full models of each outcome are reported below. Model comparison statistics can be found in S1 Table, while credible intervals and regression coefficients for each model can be found in S2 Table (politics and race) and S3 Table (politics and behavioral economics). Because participants were allowed to select more than one race in the survey, race was dummy-coded into a set of binary categorical variables, with 0 indicating that the participant did not identify as a given race and 1 indicating that they did. Gender remained categorically coded with female as the reference class. Effect sizes are reported in terms of mean and credible interval.

### Self judgments

**Fig 1:**
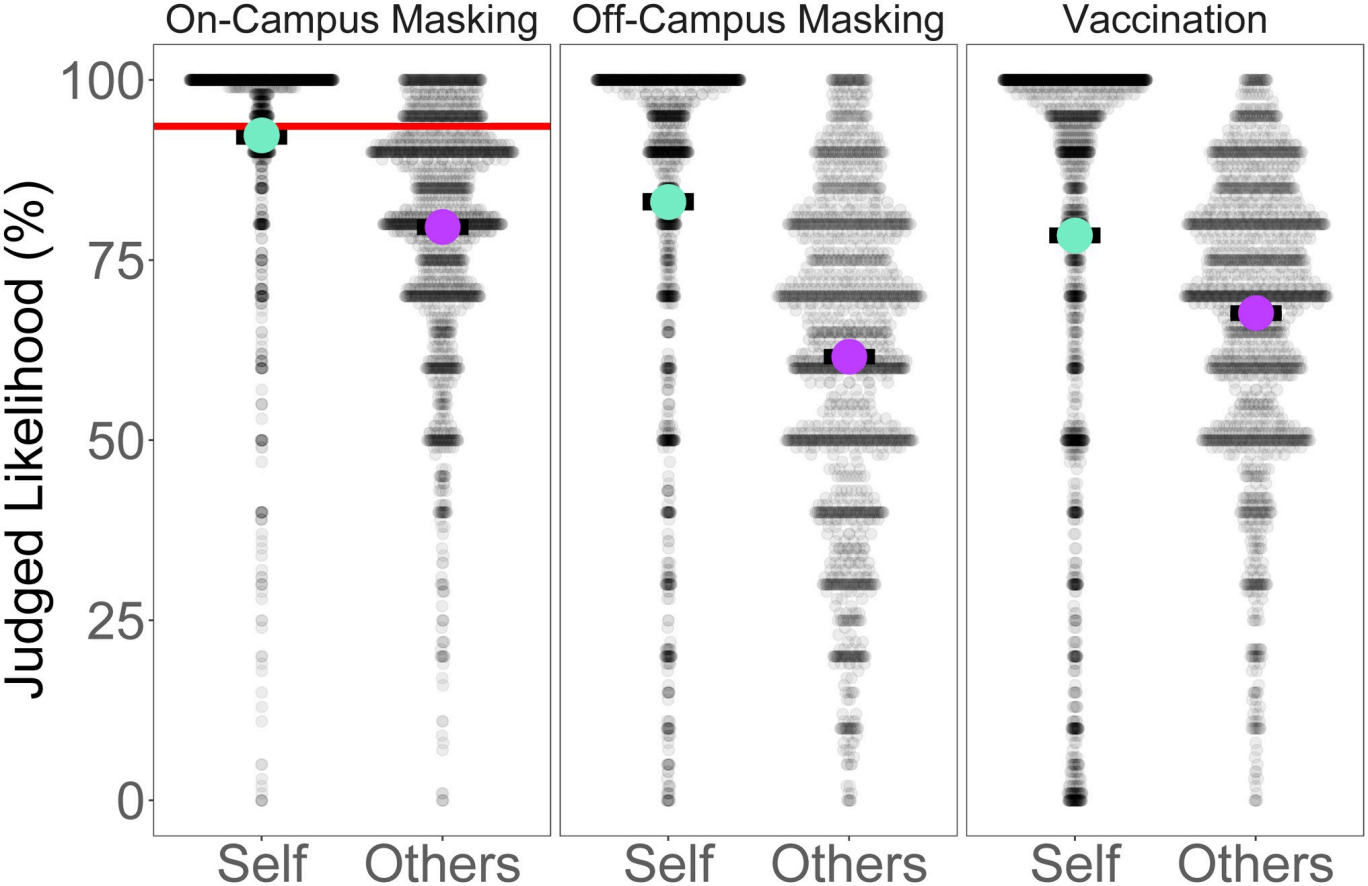
Self-Reported intent and estimates of others’ intent per outcome. Semi-transparent dots represent individual participant estimations for each category, with more opaque dots representing estimates endorsed by more participants. The larger (colored) dots represent the posterior distribution means for each outcome, while the black bars represent the 95% credible intervals around these means.

Fig 1 plots the distribution of the likelihood judgments that the participant themselves (Self) or other students wore a mask on campus, wore a mask off campus, and would seek a vaccine. Focusing first on participants’ judgments about their own behaviors and intentions (self), on average participants reported a 92% (*SD* = 17) chance that they wore a mask while on campus. This corresponded with our direct observation of the rate of mask-wearing on campus, which was 90%. Respondents reported a lower likelihood, but still more likely than not, to have worn a mask off-campus (*M* = 83.2, *SD* = 25.1) (*b* = −0.79 [−1.13, −0.44]). On average, participants also reported a lower likelihood (compared to masking on campus) to get vaccinated but were still more likely to seek a vaccine than not (*M* = 78.4, *SD* = 30.3) (*b* = 2.81 [2.51, 3.12]).

### Other judgments

Turning to judgments about other students’ behaviors and intentions, similar to the self-judgments, on-campus masking estimates were higher (*M* = 79.60, *SD* = 17.10) (*b* = 8.70 [8.12, 9.26]) and off-campus masking estimates were lower (*M* = 61.56, *SD* = 22.28) (*b* = −4.83 [−5.40, −4.27]) in reference to vaccination estimates (*M* = 67.64, *SD* = 19.67). However, the estimates of other’s intent to vaccinate were much closer to estimates of others’ off-campus masking than on-campus masking. Also note that estimates of others’ on-campus masking were, on average, 14% lower than directly observed rates.

Comparing the self-judgments with the judgments about other students’ behaviors and intentions reveals false uniqueness. Across all three judgments, respondents judged others as less likely to engage or have engaged in health behavior than themselves (*b* = −7.29 [−7.69, −6.90]). Compared to vaccination, participants estimated smaller differences between themselves and others for on-campus masking (*b* = 1.30 [0.73, 1.88]) and larger differences for off-campus masking (*b* = −3.21 [−3.77, −2.65]).

### Consensus

In our study, we also had a set of questions that allowed us to assess how widely participants assumed their estimates of others’ health behavior were shared (see Consensus measure in Methods). On average, we found some strong evidence of consensus in terms of the judgments of other students. We found that participants’ own estimations of others’ behavior were associated with the degree of consensus they expected, such that those who assumed more students had or would engage in a COVID-19 preventative behavior thought that perception was shared more widely (*b* = 0.49 [0.23, 0.76]). Compared to estimates of on-campus masking, assumed consensus for estimates of vaccination was credibly lower on average (*b* = −1.47 [−2.42, −0.51]); on- and off-campus masking were essentially treated the same (*b* = −0.63 [−1.54, 0.28]). In general, as participants estimated a greater chance of engaging in a COVID-19 preventative behavior, they reported a greater degree of consensus. This relationship between participants’ estimates was most pronounced for vaccination estimates (*b* = 0.51 [0.07, 0.96]).

### Individual differences

**Fig 2:**
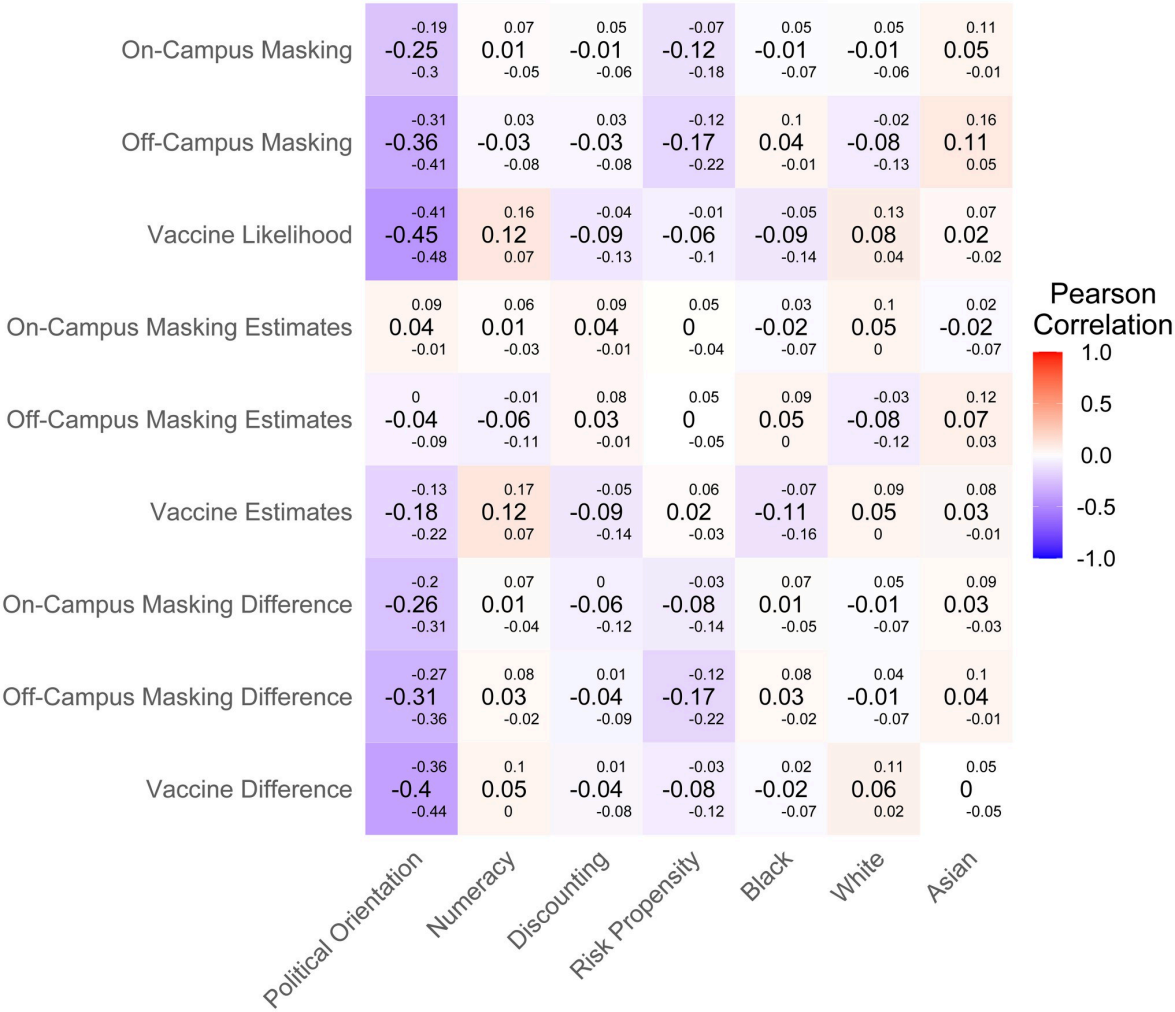
Zero-order correlations of outcome measures (behavioral estimations) and individual difference predictors. Both the Pearson correlation value and 95% credible intervals for the true value are shown. Darker red panels indicate stronger positive correlations, while darker blue panels indicate stronger negative correlations.

Fig 2 displays the zero-order correlations between outcomes and predictors in our sample, without factoring any variables out. Across different behaviors, political orientation showed a consistent negative correlation with self-reported rates and estimates of self-other differences, although its associations with estimates of others were less even. Other credible correlations were weaker, inconsistent across types of outcome measure, or both.

To account for overlapping variance among the predictors, we conducted three separate regression analyses for each of the COVID-related behaviors in our study, asking what individual difference measures predicted the estimated likelihood that individuals had or would engage in the behavior, their estimated likelihood that others had or would engage in the behavior, and the difference between self and other estimates. Table 1 lists the regression coefficients for each regression.

**Table 1:** Regression coefficients and CIs of predictors for each outcome.

*Behavior*	*On-Campus Masking*			*Off-Campus Masking*			*Intent to Vaccinate*		
Mean [HDI]	*Self*	*Other*	*Difference*	*Self*	*Other*	*Difference*	*Self*	*Other*	*Difference*
***Political Pref***.	**-0.176 [-0.238, -0.116]**	-0.021 [-0.045, 0.003]	**-0.151 [-0.204, -0.098]**	**-0.200 [-0.246, -0.153]**	0.019 [-0.004, 0.043]	**-0.145 [-0.182, -0.107]**	**-1.102 [-1.212, -0.990]**	**-0.082 [-0.103, -0.060]**	**-1.018 [-1.123, -0.915]**
*Discounting*	0.004 [-0.063, 0.072]	0.016 [-0.010, 0.041]	-0.030 [-0.087, 0.028]	-0.016 [-0.070, 0.039]	0.016 [-0.011, 0.042]	-0.008 [-0.052, 0.035]	**-0.174 [-0.295, -0.053]**	**-0.039 [-0.062, -0.016]**	**-0.140 [-0.252, -0.028]**
*Numeracy*	**0.128 [0.001, 0.255]**	0.048 [-0.004, 0.099]	0.097 [-0.012, 0.205]	-0.028 [-0.131, 0.074]	-0.048 [-0.101, 0.005]	0.030 [-0.052, 0.111]	0.188 [-0.053, 0.427]	**0.079 [0.032, 0.125]**	0.116 [-0.113, 0.344]
*Riskiness*	-0.031 [-0.097, 0.035]	-0.004 [-0.029, 0.021]	0.003 [-0.055, 0.061]	**-0.080 [-0.132, -0.029]**	0.008 [-0.017, 0.033]	**-0.093 [-0.135, -0.051]**	0.056 [-0.058, 0.169]	0.017 [-0.006, 0.039]	0.008 [-0.101, 0.118]
*Age*	0.023 [-0.036, 0.081]	0.001 [-0.010, 0.012]	-0.011 [-0.061, 0.040]	-0.016 [-0.046, 0.013]	0.000 [-0.011, 0.011]	-0.007 [-0.030, 0.017]	-0.019 [-0.016, 0.028]	-0.009 [-0.018, 0.000]	-0.001 [-0.047, 0.043]
*Male*	-0.241 [-0.542, 0.057]	0.012 [-0.110, 0.135]	**-0.284 [-0.547, -0.024]**	-0.241 [-0.483, 0]	0.045 [-0.078, 0.169]	**-0.235 [-0.425, -0.044]**	**0.915 [0.341, 1.483]**	**0.190 [0.080, 0.300]**	**0.703 [0.167, 1.238]**
*Non-binary*	0.245 [-1.220, 1.696]	-0.298 [-0.668, 0.076]	1.084 [-0.193, 2.340]	0.898 [-0.025, 1.834]	-0.234 [-0.633, 0.160]	**1.352 [0.618, 2.073]**	0.683 [-1.099, 2.469]	-0.112 [-0.467, 0.247]	0.689 [-0.995, 2.376]
*Black*	-0.486 [-1.328, 0.341]	0.104 [-0.190, 0.397]	-0.506 [-1.234, 0.235]	0.290 [-0.355, 0.933]	0.182 [-0.125, 0.494]	-0.038 [-0.542, 0.468]	**-2.406 [-3.811, -1.011]**	**-0.480 [-0.752, -0.205]**	**-1.812 [-3.127, -0.496]**
*White*	0.068 [-0.433, 0.569]	0.147 [-0.049, 0.339]	-0.107 [-0.549, 0.345]	-0.248 [-0.678, 0.183]	-0.126 [-0.324, 0.074]	0.001 [-0.333, 0.339]	**1.051 [0.156, 1.940]**	0.116 [-0.061, 0.294]	**1.039 [0.182, 1.904]**
*Asian*	0.027 [-0.543, 0.582]	-0.062 [-0.280, 0.156]	-0.009 [-0.524, 0.498]	-0.051 [-0.563, 0.455]	0.104 [-0.119, 0.328]	-0.021 [-0.432, 0.377]	0.802 [-0.195, 1.794]	0.123 [-0.075, 0.322]	0.685 [-0.279, 1.638]
*Pacific Islander*	-0.488 [-2.140, 1.156]	0.013 [-0.947, 0.976]	-0.302 [-1.725, 1.155]	-0.244 [-1.747, 1.283]	-0.292 [-1.301, 0.721]	0.029 [-1.173, 1.244]	1.654 [-3.023, 6.282]	-0.591 [-1.489, 0.296]	2.398 [-2.070, 6.884]
*American Indian*	0.264 [-0.677, 1.211]	-0.169 [-0.609, 0.265]	0.004 [-0.824, 0.829]	0.258 [-0.652, 1.174]	-0.106 [-0.560, 0.348]	-0.036 [-0.769, 0.695]	-0.632 [-2.733, 1.437]	0.194 [-0.212, 0.602]	-0.709 [-2.675, 1.249]

Coefficients are reported in the log odds scale. Coefficients for the full model can be found in the main paper. All dependent variables were run simultaneously in the same regression. Credible effects are reported in bold.

The regressions reveal that even when other predictors had been factored out, political preference was the most consistent predictor, and remained negatively associated with mask-wearing on-campus, mask-wearing off-campus, and vaccine seeking. The negative association was also present for judgments about the likelihood that other students had or would engage in the same behaviors. These negative associations imply that participants who identified as more conservative tended to estimate lower rates of health behavior in both themselves and others. For every unit towards conservatism on the political preference scale, the odds that an individual had worn a mask on campus decreased by approximately 16% [11%, 21%]; the odds that they had worn a mask off-campus decreased by 18% [14%, 22%]; and the odds that they would seek a vaccine decreased by nearly 67% [63%, 70%]. Political preference was also associated with perceived consensus for attitudes: More conservative participants assumed lower consensus for their estimations of others, although the effect of political preference was small (*b* = −0.18 [−0.32, −0.03]) and did not vary by behavior.

Some additional predictors proved credible only with certain behaviors. Discounting was negatively associated with intent to vaccinate, indicating participants who placed less worth on future rewards saw both themselves and others as less likely to vaccinate. For every unit increase in discounting, the odds of seeking a vaccine decreased by 16% [5%, 26%]. Numeracy, meanwhile, was positively albeit weakly related to likelihood to mask on-campus and and estimations of others’ vaccination rates; each unit increase in numeracy corresponded to a 14% [0.1%, 29%] increase in odds of masking on-campus and an 8% [3%, 13%] increase in odds of estimating others had masked more on campus. Risk taking proclivity was negatively associated with masking off-campus, but surprisingly not with any other behavioral outcome, indicating that personal attitudes towards risk were less relevant in on-campus judgments. For every unit increase in self-reported riskiness, the odds of wearing a mask off-campus decreased by 8% [3%, 12%].

In terms of demographics, participants identifying as Black were less likely to have an intent to vaccinate and to believe others would. However, they were no less likely to engage in any other safety measures. In our sample, men were approximately 150% [41%, 341%] more likely to express an intent to vaccinate than women and 20% [8%, 35%] more likely to assume that other students would also vaccinate. However, the opposite was true for judgments of off-campus masking, and there were no gender differences in on-campus masking.

**Fig 3:**
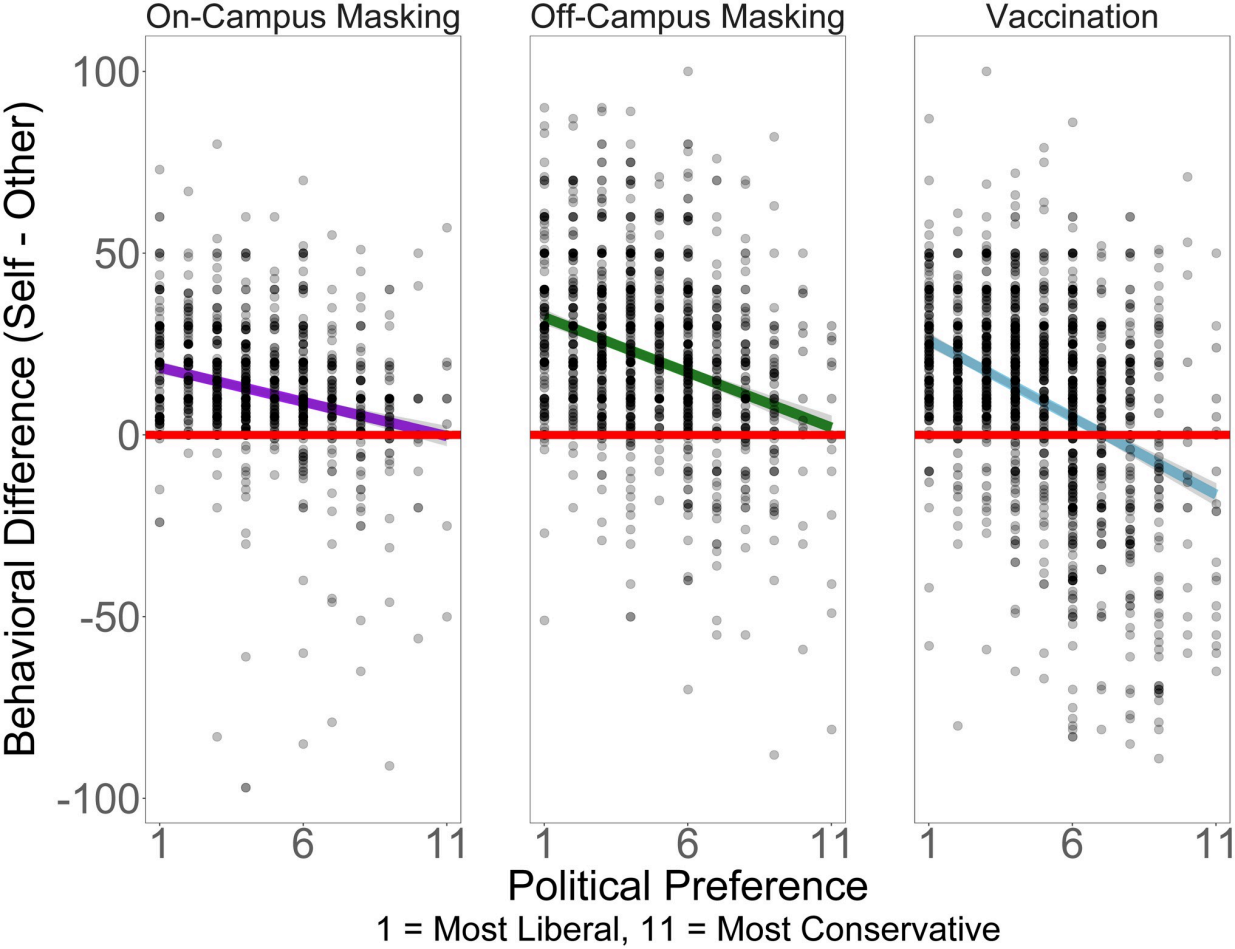
Plots of self-other differences for each health behavior outcome as a function of political preference. Higher values of x indicate more conservative political preference. (a) Association with on-campus masking (*β* = -1.88 [-2.29, -1.47]). (b) Association with off-campus masking (*β* = -3.04 [-3.53,-2.55]). (c) Association with vaccination (*β* = -4.24 [-4.72, -3.78]).

Next, we asked if any of the individual differences were associated with the difference between participants’ judged likelihood of engaging in the COVID-19 preventative behavior and their judged likelihood of others engaging in the same behavior. Again, the most consistent correlate was political preference. Fig 3 plots the difference between self and other judgments for each of the three COVID-19 related behaviors as a function of political preference. The plot reveals that the more liberal identifying respondents saw themselves as more unique while those that were more conservative saw their own behavior as more similar to others. As an index of this, we can identify the point on the political orientation scale where the difference between self and others was equal to 0. For on-campus masking (*M* = 10.92 [8.92, 13.90]), off-campus masking (*M* = 11.65 [10.03, 13.89]), and vaccination (*M* = 7.13 [6.42, 8.01]), the points of equality were all toward the conservative end of the scale.

## Discussion

The results of the study addressed all three of our key questions: (1) Individuals credibly assumed that others practice COVID-19 health behaviors at lower rates than they reported for themselves; (2) individuals falsely viewed their own behavior as more unique, with more liberal participants assuming more false uniqueness; and (3) of the individual differences examined, political preference had the most consistent association with these estimations, their differences, and false consensus associated with them. Generally, individuals within the study estimated others as less likely to engage in protective behavior than themselves, regardless of the nature of that health behavior. The more likely participants thought others were to follow protocol, the more they assumed others would make the same estimates.

The fact that more liberal participants reported higher false uniqueness was in retrospect not particularly surprising, as earlier studies had reached the same conclusion [17, 27]. What is more interesting is the ubiquity of false uniqueness throughout the sample. All but the most conservative participants assumed that they were unique in following health and safety guidelines: In estimating off-campus masking, for instance, the political level at which participants would have been perfectly calibrated was past the survey’s maximum level of conservativeness. From these results, it seems that both liberal and conservative participants saw others’ health behaviors as more conservative-aligned than their own. One important aspect to note regarding the false uniqueness in this study was that, at least in the context of mask-wearing, it was indeed *false*. By collecting direct observational data, we were able to check whether student estimates corresponded to actual rates of behavior on-campus. Compared to the data we collected, participants underestimated how frequently others wore masks on campus, but estimated their own rates much more closely to what was observed. This could indicate that participants did not rely on observations for their own estimations, instead going off more general assumptions about their fellow students [12]. While it is important to note that these data only relate to one health behavior out of the three examined, this addition affirms that assumed norms of mask-wearing did not match behavioral norms.

Multiple explanations could account for the ubiquity and direction of false uniqueness in our sample. Early politicization of COVID-19 and messages from national leadership lambasted COVID-19-related safety measures; particularly in the early days of the study, this might have influenced both individual opinions and the reference groups our participants turned to for their estimations of others [59–61]. Another potential implication of the findings is that, in this population, COVID-19 health behavior was seen as morally desirable. Health behavior can be moralized either individually, through personal experience or deliberation, or societally, as a means of mitigating what is seen as collective harm [62]. Research suggests that this process has already occurred with COVID-19 health behavior, at least individually [63, 64]. It is worth noting that, going off the assumption that false consensus and uniqueness are both motivated by the need for social validation [26], mask-wearing was implicitly seen as socially desirable by both liberal and conservative participants, despite aggressive nationwide messaging to the contrary. At the time of the study, the University of Kansas still enforced a mask mandate on-campus; while it did not specifically frame mask-wearing as a moral action, the policies could have contributed to moralization of protective behavior among the student body, thereby motivating false uniqueness.

Other factors may also have influenced the omnipresent false uniqueness we found. The study had participants report their own rates of behavior first. [27] found that participants who first reported their own rates of behavior or belief used those rates as an anchor, contrasting others’ behaviors from their own; however, the same study was inconclusive as to whether this actually affected rates of false consensus. Therefore, we do not believe this ordering significantly affected these results. Furthermore, recall that those in the actual majority were more likely to see their own behavior as unique [26]. Our direct observational data indicates that those who were highly likely to wear masks were in this majority. Directly observed rates of mask-wearing were on par with self-reports and much higher than estimations of others. Although these observations were taken relatively early into the pandemic, more recent international research on masking and distancing fatigue indicates that even now, individuals are largely willing to continue such behaviors [65]. The politicization of the pandemic might have obscured this majority, but in this sample, actual rates of health behavior did not seem to suffer from it.

Our investigations into individual differences yielded mixed results. Prior research implicated political preference as a robust predictor of COVID-19-related health behavior, even overriding perceptions of risk and other salient factors [43]. This study corroborated those results. More surprising was the lack of any other consistent, credible associations between COVID-19 preventative health behaviors and individual differences. No other individual difference was consistently associated with all three health behaviors. Nevertheless, some sporadic associations found beyond political preference have explanations rooted in prior theory; because numeracy is associated with ability to perceive risk, higher numeracy should indicate greater willingness to engage in protective measures in a risky scenario [45]. Since greater discounting, meanwhile, is associated with a more near-focused mindset, a negative association with something that was not available at the time and would primarily provide protection in the long run was also expected [54]. However, in both cases, effect sizes were relatively weak and did not generalize across outcomes, meaning that it is unclear whether these associations generalize to other health contexts and scenarios. The associations between COVID-19 preventative health behaviors and race were also limited. Participants identifying as Black reported credibly lower intent to vaccinate and more negative self-other differences, while participants identifying as White reported credibly higher intent to vaccinate and more positive self-other differences; no other associations with race were found. Even though race and political preference can be drawn upon to construct in-groups and out-groups, only political preference was associated with estimations of others and the self. The stark association between political preference and behavioral health reinforces the idea that ingroup membership translates not only to individual behavior but also perceptions of others’ behavior [66].

Despite its scope, this study has some limitations. While our sample was quite large (over 1,700 students), the demographic scope of that sample was limited; only University of Kansas students responded to the survey, meaning that the perspectives on display are those of college-educated individuals from the Midwest. Conducting the study on other individuals who simply live in Lawrence, Kansas (the location of the University of Kansas), or those elsewhere in the country could yield different results. Furthermore, few participants identified as non-White, meaning that true population effect sizes of race might be obscured by the demographics of our study. In addition, because we did not look at the composition of our participants’ social circles [15], the study’s setup left it difficult to disentangle the motivations or processes underlying self-other calibrations. A future study in this vein could delve deeper into mechanisms such as selective exposure and behavioral motivation, both of which were only implied here. Finally, our estimates were taken before any vaccine had reached wide circulation, and events related to vaccines in the interim could affect the outcomes reported here. For instance, cases of side effects from COVID-19 vaccines were not brought into the public eye until after the study period. Direct observational data of vaccination would account for this, although it would be more difficult to collect than direct observation of mask-wearing.

## Conclusion

While, by many accounts, there appears to be a light at the end of the tunnel to COVID-19 and the pandemic, the results of this study are informative for future pandemics and other scenarios. Here, we provide an example of how social cognition operates within a given community in response to a pandemic event and whether that social cognition is associated with people’s own health behaviors. False uniqueness and false consensus can both apply across broad ranges of behavior, and knowing actual norms for health and safety can guide people towards safer decisions for themselves. Understanding the drivers of differences in self-other perceptions can allow us to predict how individuals will actually respond in extreme scenarios, including pandemics and everyday decision-making. Going further, acknowledging and working with those responses can lead to better health and safety not just for individuals, but for their community and society.

## Supporting information

S1 Table Model comparisons for outcome measures. Models were compared on least squared error differences and standard deviation differences from the best fit models. Best fit models are indicated in bold for each category.
(ZIP)

S2 Table Regression coefficients for the politics and race model. Coefficients are reported in the log odds scale. Coefficients for the full model can be found in the main paper. All dependent variables were run simultaneously in the same regression. Credible effects are reported in bold.
(ZIP)

S3 Table Regression coefficients for the behavioral economics model. Coefficients are reported in the log odds scale. Coefficients for the full model can be found in the main paper. All dependent variables were run simultaneously in the same regression. Credible effects are reported in bold.
(ZIP)
