# Supplementary material for: I’m wearing a mask, but are they?: Perceptions of self-other differences in COVID-19 health behaviors
Source: PLoS One. 2022 Jun 6;17(6):e0269625. doi: 10.1371/journal.pone.0269625 (PMC9170093; doi:10.1371/journal.pone.0269625)
Supplement: S1 Table — Models were compared on least squared error differences and standard deviation differences from the best fit models. Best fit models are indicated in bold for each category. (ZIP) [file pone.0269625.s001.zip › S1_Table.pdf]

Table S1: **Model comparisons for outcome measures.**

		Self-Reports			Estimations of Others			Self-Other Differences		
		Behavioral	Race/Politics	Complex	Behavioral	Race/Politics	Complex	Behavioral	Race/Politics	Complex
Campus Mask										
	ELS Diff.	**0.0**	-4.5	-8.8	**0.0**	-3.7	-6.2	**0.0**	-3.8	-3.5
	SD Diff.	**0.0**	.3	3.8	**0.0**	2.9	2.9	**0.0**	3.4	3.4
Lawrence Mask										
	ELS Diff.	**0.0**	-4.6	-4.9	-0.3	**0.0**	-7.4	**0.0**	-13.5	2.8
	SD Diff.	**0.0**	3.7	4.9	3.9	0.0*	3.0	**0.0**	4.9	4.9
Vaccination										
	ELS Diff.	-5.2	-1.8	**0.0**	-16.6	-14.2	**0.0**	-1.2	**0.0**	-0.8
	SD Diff.	5.4	4.5	**0.0**	7.8	6.8	**0.0**	4.9	**0.0**	3.9

Models were compared on least squared error differences and standard deviation differences from the best fit models. Best fit models are indicated in bold for each category.
