# Supplementary material for: I’m wearing a mask, but are they?: Perceptions of self-other differences in COVID-19 health behaviors
Source: PLoS One. 2022 Jun 6;17(6):e0269625. doi: 10.1371/journal.pone.0269625 (PMC9170093; doi:10.1371/journal.pone.0269625)
Supplement: S3 Table — Coefficients are reported in the log odds scale. Coefficients for the full model can be found in the main paper. All dependent variables were run simultaneously in the same regression. Credible effects are reported in bold. (ZIP) [file pone.0269625.s003.zip › S3_Table.pdf]

Table S3: **Regression coefficients for the Behavioral Economics model.**

Behavior	*On-Campus Masking*			*Off-Campus Masking*			*Intent to Vaccinate*		
Mean [HDI]	*Self*	*Other*	*Difference*	*Self*	*Other*	*Difference*	*Self*	*Other*	*Difference*
Political Pref.	**-0.184 [-0.243, -0.126]**	**0.024 [0.001, 0.046]**	**-0.167 [-0.219, -0.116]**	**-0.219 [-0.264, -0.173]**	-0.019 [-0.043, 0.004]	**-0.165 [-0.202, -0.128]**	**-1.063 [-1.170, -0.957]**	**-0.070 [-0.091, -0.049]**	**-0.989 [-1.089, -0.089]**
Discounting	0.003 [-0.062, 0.068]	0.016 [-0.010, 0.041]	-0.035 [-0.091, 0.023]	-0.013 [-0.067, 0.039]	0.021 [-0.005, 0.047]	-0.012 [-0.054, 0.031]	**-0.193 [-0.313, -0.072]**	**-0.040 [-0.063, -0.018]**	**-0.157 [-0.271, -0.045]**
Numeracy	0.101 [-0.020, 0.222]	0.050 [-0.001, 0.100]	0.060 [-0.048, 0.168]	-0.038 [-0.137, 0.060]	-0.037 [-0.090, 0.016]	0.001 [-0.078, 0.080]	**0.286 [0.057, 0.522]**	**0.098 [0.052, 0.144]**	0.197 [-0.023, 0.417]
Riskiness	-0.049 [-0.113, 0.015]	-0.004 [-0.029, 0.021]	-0.014 [-0.071, 0.042]	**-0.092 [-0.143, -0.041]**	0.009 [-0.016, 0.034]	**-0.112 [-0.153, -0.071]**	0.070 [-0.044, 0.184]	0.019 [-0.003, 0.041]	0.015 [-0.091, 0.123]

Coefficients are reported in the log odds scale. Coefficients for the full model can be found in the main paper. All dependent variables were run simultaneously in the same regression. Credible effects are reported in bold.
